# Objective and automated protocols for the evaluation of biomedical search engines using No Title Evaluation protocols

**DOI:** 10.1186/1471-2105-9-132

**Published:** 2008-02-29

**Authors:** Fabien Campagne

**Affiliations:** 1 HRH Prince Alwaleed Bin Talal Bin Abdulaziz Alsaud Institute for Computational Biomedicine and Dept. of Physiology and Biophysics, Weill Medical College of Cornell University; Box 140; 1305 York Ave; New York, NY 10021, USA

## Abstract

**Background:**

The evaluation of information retrieval techniques has traditionally relied on human judges to determine which documents are relevant to a query and which are not. This protocol is used in the Text Retrieval Evaluation Conference (TREC), organized annually for the past 15 years, to support the unbiased evaluation of novel information retrieval approaches. The TREC Genomics Track has recently been introduced to measure the performance of information retrieval for biomedical applications.

**Results:**

We describe two protocols for evaluating biomedical information retrieval techniques without human relevance judgments. We call these protocols No Title Evaluation (NT Evaluation). The first protocol measures performance for focused searches, where only one relevant document exists for each query. The second protocol measures performance for queries expected to have potentially many relevant documents per query (high-recall searches). Both protocols take advantage of the clear separation of titles and abstracts found in Medline. We compare the performance obtained with these evaluation protocols to results obtained by reusing the relevance judgments produced in the 2004 and 2005 TREC Genomics Track and observe significant correlations between performance rankings generated by our approach and TREC. Spearman's correlation coefficients in the range of 0.79–0.92 are observed comparing bpref measured with NT Evaluation or with TREC evaluations. For comparison, coefficients in the range 0.86–0.94 can be observed when evaluating the same set of methods with data from two independent TREC Genomics Track evaluations. We discuss the advantages of NT Evaluation over the TRels and the data fusion evaluation protocols introduced recently.

**Conclusion:**

Our results suggest that the NT Evaluation protocols described here could be used to optimize some search engine parameters before human evaluation. Further research is needed to determine if NT Evaluation or variants of these protocols can fully substitute for human evaluations.

## Background

A search engine retrieves articles from a text collection (or text corpus) to best satisfy user queries. Articles that discuss material related to what the user was looking for when he formulated the query are defined as relevant. Other articles retrieved are defined as non-relevant. Defined sets of relevant and non-relevant documents make it possible to evaluate the performance of a search engine by calculating various quantitative performance measures. Such measures include Mean Average Precision (MAP), binary preference (bpref), precision at rank (e.g., P5, P10 or P20), among others. Performance measures and the traditional information retrieval evaluation paradigms have been reviewed in [1] and the reader should refer to this source for background information.

Most established evaluation methodologies commonly rely on domain experts to make relevance judgments for documents retrieved by search engines. For instance, in the evaluation paradigm used by various tracks (including the genomics track) in the annual Text Retrieval Conference (TREC), groups who participate in the evaluation share the same corpus and perform the same queries. Ranked lists of documents retrieved by each group are pooled to keep only unique documents, and these documents are evaluated by the judges. In another evaluation paradigm recently introduced [2], for each query, judges evaluate specific terms for their likelihood to be included in relevant documents (*onTopic *terms) or in non relevant documents (*offTopic *terms). Such term judgments are called TRels and are used to evaluate retrieval effectiveness.

Judging documents (and to a lesser extent judging terms) is an expensive activity that limits the scope of current search engine evaluations. For instance, current studies are limited to numbers of queries ranging from 25–100 because this is the number of queries for which the results can be judged by TREC staff–or judges funded by organizers of the TREC Genomics Track [3]– in a couple of months.

In this manuscript, we describe two approaches which can be used to evaluate the retrieval effectiveness of search engines without human judgments. The two approaches are entirely automated and rely on an objective metric of document relevance made possible by the document structure of Medline. We describe the approaches, discuss the reasons why they would be expected to correlate with human judgments; and present empirical evidence that confirms the existence of a significant correlation between performance measures obtained in the TREC Genomics Track and the results obtained with our approaches.

## Results

**Figure 1:**
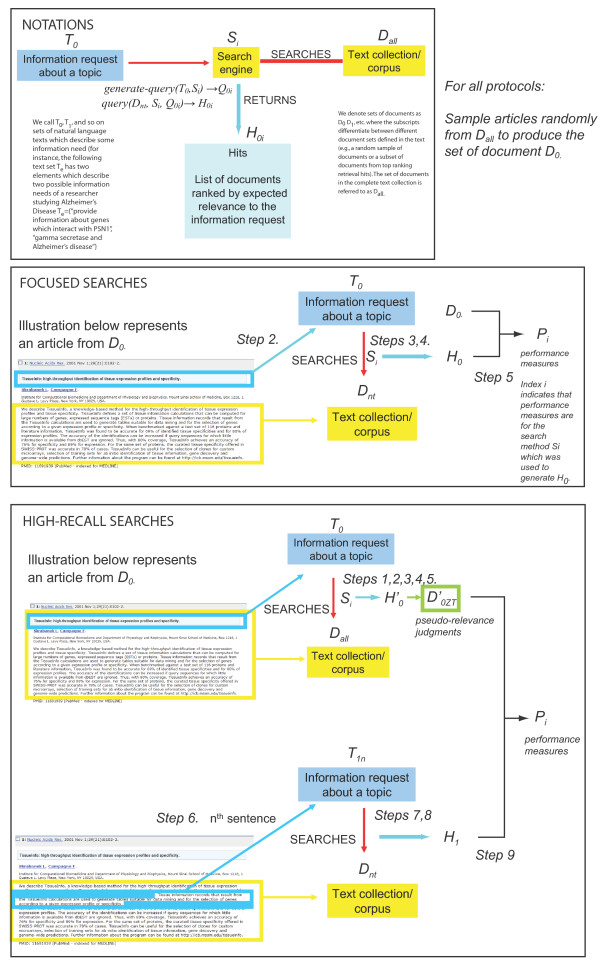
**Overview of the NT Evaluation protocols**. The three panels introduce notation used throughout the manuscript. Each protocol randomly samples documents from the text collection to produce information requests/topics. See text for a description of each protocol.

We describe two evaluation protocols in this section. The first protocol is appropriate for the evaluation of search methods for focused searches, while the second protocol is suggested for the evaluation of high-recall search methods. Figure 1 provides an overview of these two protocols. Methods common to both evaluation protocols are described in the Method section.

### Evaluating focused searches

With this protocol, we limit the evaluation to focused searches. We call focused searches requests that are expected to retrieve only one relevant document (such searches may retrieve many non relevant documents, but can retrieve one relevant document at most). In the biomedical domain, examples of focused searches include cases when the end-user is trying to locate the article that describes the discovery of the interaction between two proteins (e.g., the specific article which describes the discovery and characterization of the direct interaction between iNOS and COX2 [4]), the cloning of a specific gene, or the first demonstration that a disease is caused by mutations in a specific gene. In the web search domain, queries where users try to locate the home page of a person are also focused searches, if only one such page exists.

In the following sections of this manuscript, we denote sets of documents as *D*_0_, *D*_1_, etc. where the subscripts differentiate between different document sets defined in the text (e.g., a random sample of documents or a subset of documents from top ranking retrieval hits). The set of documents in the complete text collection is referred to as *D*_*all*_. We write *Q*_0_, *Q*_1_, and so on to denote sets of queries. We denote search engine methods as *S*_0_, *S*_1_, etc., where a search method consists of an algorithm and set of parameters. A search method *S *can process a set of queries *Q *over a document collection *D *and produce a ranked list of document hits *H *for each query in *Q*. The query operation is summarized as *query(D, S, Q) *→ *H*.

We call *T*_0_, *T*_1_, and so on sets of natural language texts which describe some information need (for instance, the following text set *T*_*e *_has two elements which describe two possible information needs of a researcher studying Alzheimer's Disease *T*_*e *_= {"provide information about genes which interact with PSN1", "gamma secretase and Alzheimer's disease"}. In TREC, *T *sets would represent the set of narratives for the topics used in a track. We define the operation *produce-query(T, S) *→ *Q *as the transformation of a set of text into a set of queries suitable for search method *S*. This notation abstracts procedures that range from completely automatic to fully manual where an expert translates an information need expressed as an element of *T *into a well-formed query for the search method *S*. We write *evaluate(H, D) *→ *P *as the process of scoring hits *H *against relevance judgments where all documents of *D *are considered relevant. A perfect search method would produce hits *H *where all the *k *documents in *D *appear in the *k *top ranks. The resulting vector *P *has one element per performance measure that is being scored. The procedure *evaluate *can therefore produce a variety of performance measures (for instance MAP (mean average precision), bpref (binary preference), or reciprocal rank).

To evaluate focused searches, we leverage the structure of Medline records. Most Medline records contain both title and abstract of the article referenced by the record. Further, most authors carefully craft the title of an article to summarize the content of the abstract. Briefly, we construct a document collection where titles have been removed and only abstracts are indexed (we call this document collection *D*_*nt*_, for documents-no-title), and ask how well the title of an article can retrieve the corresponding abstract.

More precisely, we collect a random sample of documents from the original text collection, thereafter denoted *D*_0_.

Step 1. *extract-random-sample(D*_*all*_*) *→ *D*_0_

We extract the titles of the articles in *D*_0 _and call the set of these titles *T*_0_:

Step 2. *extract-title(D*_0_*) *→ *T*_0_

We now retrieve documents from *D*_*nt *_using each element of *T*_0 _as a query. Formally, we perform the steps:

For each method *S*_*i *_under evaluation, do:

Step 3. *generate-query(T*_0_, *S*_*i*_*) *→ *Q*_*0i*_

Step 4. *query(D*_*nt*_, *S*_*i*_, *Q*_*0i*_*) *→ *H*_*0i*_

We score the *H*_*0i *_hits with traditional measures of information retrieval performance using *D*_0 _as the relevance document set. Formally,

Step 5.* evaluate(H*_*0i*_, *D*_0_*) *→ *P*_*i*_

This overall focused evaluation strategy produces evaluation measures which indicate how well a search method *S*_*i *_can identify the abstract of an article using the title of the article as the query, when the title is absent from the text collection.

This strategy can be seen as mimicking the scenario where a user is trying to locate an article in Medline for which the user only remembers some keywords about what the article was about. Indeed, the title of an article is likely to contain similar keywords to those found in the abstract, but is also unlikely to contain exactly the same words as found in the abstract because authors try to avoid repetitions.

The soundness of this evaluation strategy should be self-evident since the title of an article is clearly relevant to the abstract of the same article (if that were not the case, the title would not match the relevant abstract for any of the method evaluated). However, is this evaluation scheme inadequate because it evaluates such a trivial problem that most information retrieval approaches will always find the correct answer in the first document retrieved? If so, comparisons among information retrieval approaches would be impossible because each method would have the best performance score. Table 1 show that this is not the case. This table lists the mean reciprocal rank obtained by different search approaches and measured over Medline with the focused evaluation search for a query set of 1,000 titles. Mean reciprocal rank is ideal to measure the performance of a focused search because it averages the inverse of the rank of the relevant document averaged over each query. A value of 1 would indicate that the relevant document was always found at rank 1 in the list of retrieved result, for each query. Values in Table 1 range from 0.493 to 0.580. The span of the values shows that the evaluation methodology for focused searches can rank search approaches by performance. (Details of the approaches are given in Method Details).

**Table 1:** Mean Reciprocal Rank for 1,000 queries (focused search evaluation) measured for 13 search methods.

**Mean Average Rank**	**Mean Reciprocal Rank**	**Search Method**	**Stemmer**	**Twease Slider Position**
2.028	0.493	BM25ec	Porter	0
2.054	0.487	BM25ec	Paice-Husk	0
1.787	0.560	BM25ec	None	0
1.750	0.571	BM25ec	None	20
1.724	0.580	BM25ec	None	40
1.732	0.577	BM25ec	None	60
1.732	0.577	BM25ec	None	80
1.728	0.579	BM25ec	None	100
1.737	0.576	BM25ec	None	120
1.752	0.571	BM25ec	None	140
1.755	0.570	BM25ec	None	160
1.760	0.568	BM25ec	None	180
1.767	0.566	BM25ec	None	200

### Evaluating high-recall searches

The strategy that we present here aims to evaluate approaches for high-recall searches. In contrast to focused searches, high-recall searches are expected to retrieve more than one, and potentially many, relevant documents for each query. This is the search scenario that is typically evaluated in TREC (for instance in the ad hoc task of the TREC terabyte track). We propose a simple extension to the focused search evaluation strategy to evaluate high-recall searches. We start as for the focused search evaluation and produce *D*_*nt*_, *D*_0 _and *T*_0_.

The following step uses a search engine *S*_*ref *_to query *D*_*all *_with *T*_0 _and produce *H'*_0 _hits:

Step 1. *generate-query(T*_0_, *S*_*ref*_*) *→ *Q*_0_

Step 2. *query(D*_*all*_, *S*_*ref*_, *Q*_0_*) *→ *H'*_0_

*H'*_0 _is produced by searching the document collection with titles, while *H*_0 _was produced by searching *D*_*nt*_(collection without titles).

We keep the *k *highest scoring hits from *H'*_0 _to produce *H'*_0 [*1..k*] _(in this manuscript, we used k = 1,000).

Step 3.*best-scores(H'*_0_, *k*) → *H'*_0 [*1..k*]_

We evaluate the Z-score for each document in *H'*_0 [*1..k*]. _That is, if query Q_0p _matches documents d_0pq _with score s_0pq_, we calculate:

${\text{Z-score(d}}_{{\text{0}}_{\text{pq}}}\text{)}=\;\frac{{\text{s}}_{{\text{0}}_{\text{pq}}}\text{- }\bar{{\text{s}}_{{\text{0}}_{\text{q}}}}}{\sigma }$, where $\;\bar{{\text{s}}_{{\text{0}}_{\text{q}}}}$ is the average value of ${\text{s}}_{{\text{0}}_{\text{pq}}}$ over *H'*_0 [*1..k*]_, and σ the standard deviation of ${\text{s}}_{{\text{0}}_{\text{pq}}}$ over *H'*_0 [*1..k*]. _We select hits with a Z-score greater or equal to a threshold (ZT) to produce *H'*_*0ZT*_. We then reduce *H'*_*0ZT*_to the set of documents *D'*_*0ZT*. _We used ZT = 2 in this study. These steps can be summarized as follow:

Step 4.* best-Z-score(H'*_0 [*1..k*]_, *ZT*) → *H'*_*0ZT*_

Step 5. *documents(H'*_*0ZT*_) → *D'*_*0ZT*_

Our evaluation strategy stands on the assumption that *D'*_*0ZT*_ can be used as a set of relevant documents when evaluating a query over *D*_*nt*_.

Since we have used *T*_0 _to produce a set of relevant documents, we need an independent set of texts (we will denote this text *T*_1_) that can be used to evaluate retrieval effectiveness against *D'*_*0ZT*_. We chose to construct this text with the n^th ^sentence of each abstract in *D*_0_. In this manuscript, we have used the third sentence of each abstract in *D*_0 _to produce the set of texts *T*_1. _We have confirmed that evaluation results are insensitive to the choice of which sentence is used when taken from the same abstract (data not shown). We proceed with *T*_1 _to query *D*_*nt *_and evaluate performance against the *D'*_*0ZT *_pseudo-relevance judgments:

Step 6. *extract-sentence(D*_0_, *n) *→ *T*_*1n*_

For each search method under evaluation *S*_*i*_, do

Step 7. *generate-query(T*_*1n*_, *S*_*i*_*) *→ *Q*_*1i*_

Step 8. *query(D*_*nt*_, *S*_*i*_, *Q*_*1i*_*) *→ *H*_*1i*_

Step 9. *evaluate(H*_*1i*_, *D'*_*0ZT*_*) *→ *P*_*i*_

**Figure 2:**
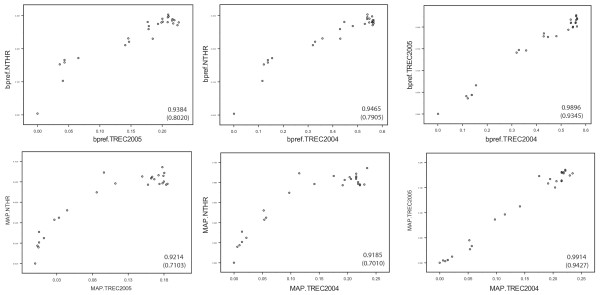
**MAP and bpref performance measures obtained by NT Evaluation and TREC evaluation**. The scatter plots compare the performance of methods measured in the NT Evaluation protocol and with TREC relevance judgments (left four plots), or compare agreement between two independent TREC Genomics Track evaluation (rightmost plots). Pearson correlation coefficients are shown in each scatter plot (values in parentheses are Spearman rank correlation coefficients). Better correlations are observed when bpref measures are compared (top row of scatter plots) vs. MAP measures (bottom row).

In contrast to the evaluation for focused searches, it is not clear *a priori *that the high-recall evaluation protocol presented here will produce performance measures that adequately reflect the retrieval performance of the methods under test. To gain insight into this question, we asked whether the results of the evaluation would correlate with the results of a traditional evaluation. Figure 2 and Table 2 present empirical results that indicate that the high-recall evaluation protocol presented in this manuscript generates performance measurements that correlate with those obtained in TREC (genomics tracks 2004 and 2005). The search methods tested are described in Table 3.

**Table 2:** Correlation coefficients for data in Figure 1. Pearson's coefficients are shown followed by Spearmans' rank coefficients in parentheses.

	**mapTREC**	**bprefTREC**	**mapNT**	**bprefNT**
**mapTREC**	1.0000	0.9291 (0.9780)	**0.9214 (0.7103)**	0.8416 (0.8057)
**bprefTREC**		1.0000	0.9560 (0.6575)	**0.9384 (0.8020)**
**mapNT**			1.0000	0.9373 (0.8307)
**bprefNT**				1.0000

**Table 3:** Search methods *S*_*i *_compared with the high-recall NT Evaluation protocol.

**Twease Slider Parameter Position**	**Tag**	**Scorer Name**	**Query Distributor**	**max Word Keep Parameter**	**TF-IDF Pseudo Relevance Feedback**	**Max New Terms Parameter**	**Top Documents To Inspect Parameter**
0	3	INTER_MATCH_DISTANCE_SCORER	DisjunctiveQueryDistributor	8	no	N/A	N/A
0	4	INTER_MATCH_DISTANCE_SCORER	ConjunctiveDisjunctiveQueryDistributor	16	no	N/A	N/A
0	5	bm25ec	DisjunctiveQueryDistributor	8	no	N/A	N/A
0	6	bm25ec	ConjunctiveDisjunctiveQueryDistributor	16	no	N/A	N/A
0	7	BM25EC2_IMD_SCORER	DisjunctiveQueryDistributor	8	no	N/A	N/A
0	8	BM25EC2_IMD_SCORER	ConjunctiveDisjunctiveQueryDistributor	16	no	N/A	N/A
0	9	bm25ec	ConjunctiveDisjunctiveQueryDistributor	8	no	N/A	N/A
0	10	INTER_MATCH_DISTANCE_SCORER(1,-1)	DisjunctiveQueryDistributor	8	no	N/A	N/A
0	11	INTER_MATCH_DISTANCE_SCORER(-1,1)	DisjunctiveQueryDistributor	8	no	N/A	N/A
0	14	INTER_MATCH_DISTANCE_SCORER(-3,1)	DisjunctiveQueryDistributor	8	no	N/A	N/A
0	15	INTER_MATCH_DISTANCE_SCORER(-2,1)	DisjunctiveQueryDistributor	8	no	N/A	N/A
20	20	BM25EC2_IMD_SCORER	ConjunctiveDisjunctiveQueryDistributor	8	no	N/A	N/A
80	21	BM25EC2_IMD_SCORER	ConjunctiveDisjunctiveQueryDistributor	16	no	N/A	N/A
160	22	BM25EC2_IMD_SCORER	ConjunctiveDisjunctiveQueryDistributor	16	no	N/A	N/A
200	23	BM25EC2_IMD_SCORER	ConjunctiveDisjunctiveQueryDistributor	16	no	N/A	N/A
20	40	bm25ec	ConjunctiveDisjunctiveQueryDistributor	16	no	N/A	N/A
80	41	bm25ec	ConjunctiveDisjunctiveQueryDistributor	16	no	N/A	N/A
160	42	bm25ec	ConjunctiveDisjunctiveQueryDistributor	16	no	N/A	N/A
200	43	bm25ec	ConjunctiveDisjunctiveQueryDistributor	16	no	N/A	N/A
20	50	bm25ec	DisjunctiveQueryDistributor	16	no	N/A	N/A
80	51	bm25ec	DisjunctiveQueryDistributor	16	no	N/A	N/A
160	52	bm25ec	DisjunctiveQueryDistributor	16	no	N/A	N/A
200	53	bm25ec	DisjunctiveQueryDistributor	16	no	N/A	N/A
20	60	bm25ec	DisjunctiveQueryDistributor	16	yes	15	15
80	61	bm25ec	DisjunctiveQueryDistributor	16	yes	10	15
160	62	bm25ec	DisjunctiveQueryDistributor	16	yes	5	15
200	63	bm25ec	DisjunctiveQueryDistributor	16	yes	15	20

The correlation observed between mapTREC and mapNT is significantly different from a random correlation (P-value = 0 two-tailed test Pearson coefficients correlation calculated with S-Plus 7.06). The same is true comparing bprefTREC and bprefNT (P-value = 0 two-tailed test). Correlation tests using ranks are also significant (P-value = 4.35 10^-5 ^using a Kendall-τ test for MAP, P-value = 2.26 10^-6 ^for bpref). See the Discussion section for a comparison of these correlation coefficients to the correlations reported in other studies.

**Figure 3:**
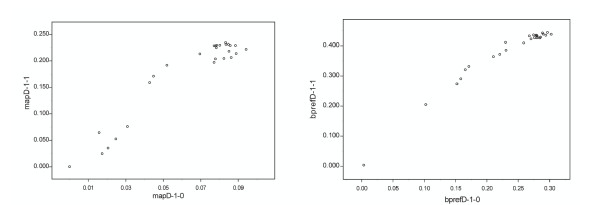
**Sensitivity of the evaluation to the search method S_ref_**. Two different search methods were used in Step 1 of the high-recall evaluation (n = 29 search methods tested). The panels show MAP and bpref agreement between these two runs. A stronger agreement is observed for bpref than for MAP (MAP/MAP correlation coefficient: 0.9540, bpref/bpref: 0.9740). These results indicate that the high-recall evaluation protocol produces performance measures which are marginally dependent on the choice of the S_*ref *_method used to perform Step 1.

We next asked to what extent the results of a high-recall evaluation were sensitive to the choice of the method *S*_*ref *_used to generate relevance judgments (see parameter *S*_*ref *_in Step 1 above). To address this question, we generated relevance judgments with a different search method than was used to produce Figure 2. Figure 3 plots how well the MAP and bpref measures agree between the two evaluations.

**Figure 4:**
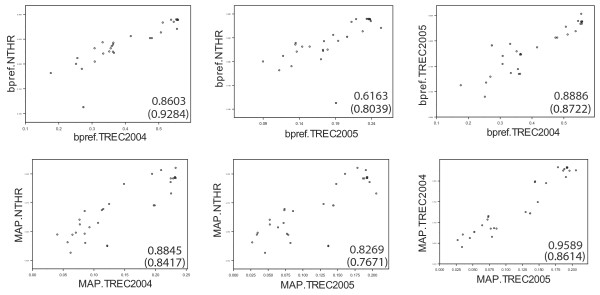
**Evaluations with a different sample of search methods**. Different search methods than used in Figure 1 were evaluated with NT evaluation and with TREC Genomics Track 2004 and 2005 relevance judgments. Pearson correlation coefficients are shown in each scatter plot (values in parentheses are Spearman rank correlation coefficients). As for the sample used in Figure 1, better correlations are observed when bpref measures are compared (top row of scatter plots) vs. MAP measures (bottom row).

In the plot shown in Figure 2, each point represents a search method under evaluation. To estimate the impact of the choice of these methods on the correlation coefficients reported in this manuscript, we performed a comparison with another set of methods (description of methods in this set is provided [in Additional File 1]). Figure 4 compares the performance of this different sample of methods, as measured on TREC 2004, 2005, and with the NT evaluation protocol. The sample of methods shown in Figure 4 also indicates a strong correlation between the results of NT Evaluation and the results of the TREC Genomics Track evaluations. In one instance the Spearman correlation coefficient is higher than when two TREC Genomics Track evaluations are compared (0.9284 for bpref TREC 2004 vs. bpref NT evaluation high-recall search compared to 0.8722 for bpref TREC 2004 vs. bpref TREC 2005). This strongly suggests that NT Evaluation produces performance estimates that can approach that of TREC Genomics Track evaluations.

**Figure 5:**
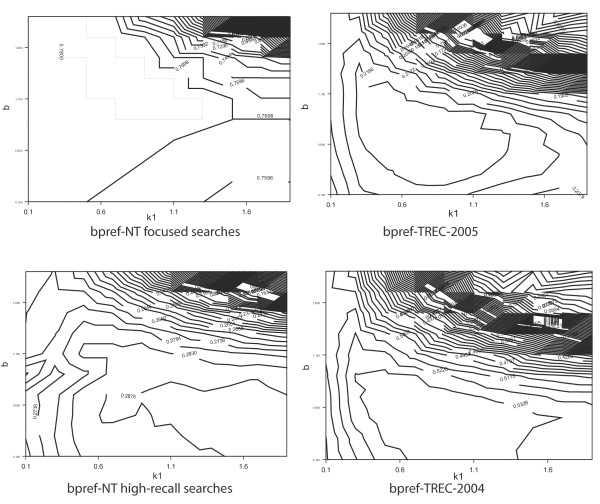
**NT Evaluation predicts favorable regions of the search parameter space**. Each contour plot shows how retrieval performance changes with the value of parameters k1 and b of the Okapi BM25 search method. The top-left plot is constructed for focused searches. The two plots on the right are constructed with TREC Genomics Track relevance judgments. The plot on the bottom left is constructed with the high-recall NT Evaluation protocol. High-recall NT Evaluation and TREC Genomics evaluations show similar performance contours with respect to parameters, suggesting that NT Evaluation can be used to select reasonable search engine parameters without human relevance judgments.

Finally, we asked if NT Evaluation could help tune the parameters of a specific search method without human judgments. Search methods often contain parameters that require tuning for each text collection. An example is the Okapi BM25 probabilistic scoring method, which accepts two parameters k1 and b. Choice of parameters has been shown to significantly affect retrieval performance in past TREC experiments. We therefore tested the ability of NT evaluation to identify favorable and unfavorable regions of the BM25 parameter space. Figure 5 shows that performance of BM25 varied with k1 and b in a similar manner when measured with bpref on TREC Genomics Track 2004, 2005, and with NT evaluation high-recall. Significantly, the contour plots produced with NT Evaluation clearly identify regions of the parameter space that yield low retrieval performance. Contours for best performance also overlap between NT Evaluation and TREC Genomics Track evaluation (compare contour bpref = 0.2830 bottom left plot with bpref = 0.5326 contour on the bottom right plot).

## Discussion

In this manuscript, we have presented NT Evaluation, two protocols to evaluate the performance of a search engine. The first protocol helps evaluate so called focused searches, i.e., those searches expected to retrieve one or a few relevant results.

### Focused vs. high-recall searches

A drawback of focused search evaluations is that it is unclear if the performance of search approaches measured for focused searches is indicative of the performance of the same approaches for searches expected to retrieve potentially many results. The question is not just academic because Büttcher et al. have recently tried to optimize search parameters for either focused searches or high-recall searches in the TREC 2006 terabyte track, and have not been able to obtain a parameter set that would perform optimally for both types of searches [5]. The results reported in [5] do not entirely establish that focused searches are intrinsically different from high-recall searches, since the difference in parameters could have been caused by artifacts introduced by the construction of the text collection (named pages have a shorter length on average in the TREC corpus used). Yet, the suspicion remains that the two types of searches may be sufficiently different that evaluating approaches with focused searches may not inform about the performance of the same approaches for high-recall searches.

Interestingly, NT evaluation for focused searches shows very different contours for the best parameter performance compared to high-recall searches, with a preference for higher values of the parameter *b *(i.e., values of *b *higher than 1 seem to be preferred, see contour of bpref value = 0.7800). A similar preference for high-values of *b *was also observed by Buttcher et al. for named page searches [5]. These differences support the hypothesis that focused searches and high-recall searches are distinct tasks that may benefit from different search parameters.

### Evaluation with random judgments

Our work aims to develop and test approaches that require minimal human assessment of the information retrieved by the search engine. While Soboroff and colleagues pioneered research in this field by considering random relevance judgments [6], it should be noted that their method is unable to rank best and worse performing systems reliably and is therefore of little practical use.

### Evaluation with TRels

Notable progress has been accomplished in this field of study with the development of the TRels approach [2]. This approach reduces human evaluation efforts to the assembly of TRels (lists of *onTopic *and *offTopic *terms for each query in an evaluation). Correlation coefficients reported for a TRel evaluation vs a TREC evaluation (MAP-tScore correlation) was 0.938 (Pearson's) and 0.746 (Kendall's-τ). Compared with a different TREC evaluation, NT Evaluation reaches correlation coefficients of 0.9214 (Pearson's) and 0.562 (Kendall's-τ). This comparison therefore suggests that TRels correlate better with TREC evaluation than NT Evaluation. However, while TRels are easier to assemble than document relevance judgments, the approach is still semi-automatic because each query must be studied to identify *onTopic *and *offTopic *terms. In contrast to TRels, NT Evaluation requires no human assessment of terms potentially present in retrieved documents. Consequently, NT Evaluation can be used to perform large scale evaluations with tens of thousands of queries. For instance, we report in this manuscript a series of evaluation runs performed with 1,000 queries over the whole set of MEDLINE abstract and titles (see Focused evaluation protocol, Table 1). This evaluation would have been impractical with the TRels approach.

### Estimates of MAP from samples of judged documents

In an elegant paper, Aslam and colleagues describe how to efficiently sample pairs of documents to produce unbiased estimates of MAP with low variance [7]. Empirical tests of this approach on TREC 8 data suggest that useful estimates can be derived with as little as 4% of the TREC judgments (corresponding to 29 judged documents per query). With 29 judged documents per query, correlation coefficients of 0.9351 and Kendall τ of up to 0.74 were obtained. Correlations improve as larger samples of judged documents are considered. With 200 judged documents per query on average, correlation coefficients reach 0.99 (Pearson) and 0.91–0.94 (Kendall τ). The authors indicate that the sampling method makes it possible to estimate the absolute value of MAP for a search method and prefer root mean square deviation to correlation coefficients. Values of MAP can vary widely for a given method when measured on different samples of topic (i.e., compare MAP values measures on the TREC genomic track 2004 and 2005 topic collections). It is therefore unclear that estimating an absolute MAP value has practical interest. Further, the variability of absolute performance values with the sample of topics considered in the evaluation is not considered by Aslam et al (i.e., MAP is estimated on TREC 8 and compared to TREC-8 judgments). Sources of variability due to varying query difficulty in the topic samples may therefore yield lower correlations than reported in the study of Aslam.

### Evaluation with Data Fusion

Data fusion is a technique where hits from different search engines are aggregated based on rank. In a recent article, Nuray and Can show that data fusion can produce pseudo-relevance judgments which correlate with TREC evaluations [8]. When fusing results from the best search engines in an evaluation, they obtain mean Spearman's correlation coefficients ranging from 0.752 to 0.854. Since the identity of the best systems is unknown before a TREC evaluation is conducted, these correlation values should be regarded as an upper-bound on the correlation that the data fusion approach can produce. Indeed, when the identity of these systems is unknown *a priori*, the approaches described in [8] achieve on average lower correlation with TREC (average correlations range from 0.527 to 0.627 depending on the data fusion technique used). Correlations obtained with the NT Evaluation protocol are above 0.7, suggesting that NT Evaluation outperforms data fusion (confirmation of this claim will require testing NT Evaluation on the same text TREC evaluations and systems as reported in [8]). Furthermore, in [8] data fusion was used with official document results from methods that participated in the TREC evaluation (results were used by TREC staff to build the pool used by the assessors). Methods included in a TREC pool are known to evaluate better on average than methods not in the pool evaluated. Our study evaluates some search methods which were related to methods in the TREC pool, and others very dissimilar. It is unclear what level of correlation would be observed with data fusion if used to evaluate mixes of in-the-pool and out-of-the-pool systems.

### Cross topic variability

A long history of TREC evaluations has shown that performance of the same approach can vary widely from one topic/query to the next, so that performance measures are now only reported on sets of queries [1]. This effect can clearly be seen in Figures 2 and 4, where performance of the same set of methods measured with TREC Genomics Track data from 2004 and 2005 yields Spearman correlation coefficients in the range 0.86–0.87. To counter the effects of topic variability on performance, TREC experiments try to sample the types of topics that users of the search engines are likely to be interested in and search for. In TREC, this is usually achieved by interviewing search engine users and asking for examples of searches that the users would perform. In the NT Evaluation protocol, however, topics used to generate queries are randomly sampled from the document collection without interviewing users. In the case of Medline, this results in search topics from basic research to clinical interests. While NT Evaluation provides no guarantee that somebody would want to perform a search corresponding to a given query used in the evaluation, there must be some level of interest in the topic since each topic is derived from an article published and indexed in Medline (also, information topics more frequently discussed in Medline are more likely to be sampled as evaluation topics).

### Subjectivity of human evaluations

TREC evaluations have also shown that assessor disagreement is common when human judges assess documents. Disagreement occurs when one assessor judged a document relevant while another assessor judged the same document non relevant to the topic. Detailed analysis of the impact of assessor disagreement showed a minimal impact on the reproducibility of performance estimates as long as all the systems tested are assessed consistently (all systems compared judged by the same set of assessors) [1]. The fact that two assessors can disagree on 30% of documents in various TREC experiments confirms the subjective nature of relevance. A recent study by Dong et al suggests that the cause of assessor disagreement may be rooted in the different background and familiarity of the judges with the material discussed in the documents [9]. The NT Evaluation approach substitutes an objective measure that is used consistently across the entire evaluation (objective in the sense that the protocol can be automated and is reproducible). Because it does not use human judgments, the NT approach can be considered not sensitive to assessor disagreement.

### NT Evaluation as yet another assessor

An alternative view suggested by a reviewer of this manuscript would be to consider the NT approach as yet another assessor. However, there are major differences between a human assessor and the protocol described in this manuscript. Most importantly, NT Evaluation cannot produce relevance judgments for arbitrarily formulated topics. Human assessors have no problem judging relevance of documents to arbitrary topics as long as the topics overlap with the assessors domain of expertise. In contrast, NT Evaluation considers only topics that can be constructed from the corpus by sampling a random set of documents (see Figure 1). Therefore, each topic is derived from a single article in the corpus. This is usually not the case in evaluations that involve human assessors. It should be noted that this difference prevents the comparison of inter-assessor agreement between NT Evaluation and traditional human relevance judgments. Indeed, there is no way to calculate statistics of assessor agreement when the set of topics 'judged' is disjoint. This is the reason why our study evaluated the correlation in overall system performance between NT Evaluation and TREC Genomics Track evaluations.

### Sensitivity to the quality of titles

Medline is a text collection where the quality of titles is high. Most articles are described with a title and an abstract such that the title accurately describes the content of the abstract. Exceptions occur, however, and the titles of some articles may not contain enough information to locate the corresponding article (i.e., consider "A productivity study", a non informative title, or an article with a title but no abstract). The NT Evaluation protocols leverage the association between title and abstract in Medline. How are the protocols affected when there are many non informative titles in the text collection? Since the focused evaluation protocol derives queries from titles, noninformative titles will fail to match the corresponding abstract. For a given method, a higher proportion of non informative titles will therefore decrease the performance of the approach. However, because the set of queries is fixed for all methods under consideration in the NT evaluation protocol, the decrease in performance will be consistent across all methods under evaluation. Higher proportions of non-informative titles in the text collection require the evaluation of more abstracts to find some that match documents, but do not affect the relative performance scores of the methods compared. The same argument can be made for the high recall NT evaluation protocol because the set of queries used is again fixed for all methods under evaluation.

### Sensitivity to the *S*_*ref *_parameter

In the first step of the high-recall NT Evaluation, we use a search method to assemble the pseudo-relevance judgments that other search methods will be judged against. This could introduce a bias in the evaluation in that the methods among *S*_*i *_most similar to *S*_*ref *_would score better than other methods. We minimize this problem by giving an unfair advantage to *S*_*ref *_(used in constructing the relevance judgment): this method is allowed to search the full text collection (*D*_*all*_, including titles), while all other methods being evaluated can only search the no-title subset of the text collection (*D*_*nt*_). Since the methods being evaluated (*Si*) only see a subset of the information, they must outperform *S*_*ref *_to retrieve the full set of pseudo relevant documents *D'*_*0ZT *_already identified by *S*_*ref *_and rank at maximum performance. We tested that this approach is effective by using two different *S*_*ref *_methods in Step 1. We used one strong method and another with about half the performance (measured on the TREC relevance judgments). The performance measures obtained for evaluated methods appeared relatively insensitive to the choice of *S*_*ref *_(correlations shown on Figure 3).

### Fusion of search engine results

A powerful method to improve retrieval effectiveness is to combine results of different search methods. Various approaches have been developed to this effect, for instance rank fusion [8], or the combination of individual method scores, as described in [10]. Fusion methods weight each method to produce a final ranking of results where each method has a given influence. Machine learning methods have also been used to learn how best to combine results from different search methods and improve search effectiveness (see [11] and references therein). Optimization of fusion parameters is an important activity that could benefit from the NT evaluation protocols. The ability to scale up the number of queries considered in the evaluation may allow the determination of general fusion parameters that work well across a variety of topics. If sufficiently large numbers of queries are evaluated (i.e., 1,000 queries or more), it may also be possible to mine the resulting data to determine which parameters will work best for specific queries or classes of queries. This type of study is currently impossible because of the cost of human relevance judgments.

### Future experiments

Our results indicate that the high-recall evaluation protocol produces performance measures that correlate with results obtained in the 2004 and 2005 TREC Genomics Track evaluations. Testing the correlation of NT Evaluations with the results of other TREC evaluations (e.g., ad hoc terabyte track which traditionally evaluates 50 topics per year and has been organized for several years) will help establish how well this protocol agrees with evaluation protocols relying on human judges. The NT Evaluation protocol described here must be adapted to noisy HTML text collections before these experiments can be conducted.

## Conclusion

We have presented two evaluation protocols designed to evaluate biomedical search engines over Medline. The protocols are fully automated and do not require human relevance judgment, but will require further validation on large non-biomedical text collection before they can be used confidently for search engine evaluation. If future evaluations confirm our findings, NT Evaluation protocols will allow scaling up search engine evaluation studies to very large number of queries. The first protocol described makes it possible to evaluate search engines when users look for one relevant document per query. The second protocol supports evaluation of searches when many relevant documents are expected. The evaluation protocols that we have described can be used to optimize the parameters of search engines for a specific corpus in the absence of preexisting relevance judgements.

## Methods

### Search Methods

Table 3 describes the search methods that were compared in this study. To obtain a sample of methods with different performances, we varied several search method parameters. Parameters varied included:

• query generation approach;

• document scoring approach and parameters (BM25ec, INTER_MATCH_DISTANCE_SCORER (IMD scorer), or BM25EC2_IMD_SCORER);

• use or not of relevance feedback (and associated parameters).

These approaches are described in the sections below. All queries were performed with MG4J 1.1.2.1 (local version derived from the official MG4J distribution 1.1.2) and the latest development version of the Twease search engine [12,13].

### Scoring approaches

BM25ec is an extension of the Okapi BM25 scoring method [14,15] presented in [12]. INTER_MATCH_DISTANCE_SCORER (IMD scorer) is a scoring approach which uses only information about the distance between matches of the query words to the document. To estimate the IMD score, minimal interval semantic [16] is used to determine the intervals of text that match the query, these sets of intervals are pruned to remove overlapping intervals (when two intervals overlap, the shorter is kept), and the gaps between these intervals are used to evaluate:

$imdScore(d)=\frac{\sum_{g:gaps\;in\;d}s*length{\left(g\right)}^{e}}{{\left(length(d)/D\right)}^{e}}$, where *length(d) *denotes the length of the document, and *D *denotes the average length of documents in the text collection. Because the IMD scorer only uses information about the density of query word matches to a document, it is not expected to do well (and was included in this study as an example of poorly performing method). We use the IMD scorer and combinations of this scorer with BM25ec in this evaluation to provide intermediate low performance search approaches. The BM25EC2_IMD_SCORER scores documents as the linear combination of scores from BM25ec and IMD: 2*BM25ec-Score + IMDScore.

### Query Generation

Query generation implements the procedure *generate-query(T*_0_, *S) *→ *Q*_0. _We used three automatic query generation algorithms: DisjunctiveQueryDistributor, ConjunctiveDisjunctiveQueryDistributor and CombinationAndThenQueryDistributor. Each algorithm first tokenizes *T*_0 _to produce a list of unique word-tokens. The list is sorted by increasing corpus frequency for each token. Assume the list of words produced is {A, B, C, D, E}, with frequency of token A in corpus less or equal to frequency of word B. DisjunctiveQueryDistributor will produce the query A|B|C, including at most maxWordKeep = 3 words in the final query. ConjunctiveDisjunctiveQueryDistributor will split {A, B, C, D, E} into two lists {A, B} {C, D, E} and produce the query (A|B)(C|D) when maxWordKeep = 2. CombinationAndThenQueryDistributor implements the query generation mechanism described in [16] and produces

(A&B&C&D), (A&B&C)| (B&C&D) |(A&C&D), (A&B)| (B&C) |(C&D)|(A&C)|..., A|B|C|D. The symbol & represents conjunction and the symbol ',' represents the "and then" query mechanism described in [16]. Our implementation is governed by maxWordKeep, the number of words to include in the query, starting with lowest frequency words, and maxInclude, the number of words per disjunctive clause in the last and then query clause generated.

### Pseudo-Relevance Feedback

Some runs were performed with pseudo-relevance feedback. Such runs are performed in two steps. The first step obtains the same hits as when no relevance feedback is used. The second step inspects *k *top documents retrieved to select *j *words. Words are selected by rank according to TF-IDF score in the *k *documents considered for feedback, and are used for scoring with a BM25 score multiplied by 1/3. This process is different from that described in [17,18] and aims to produce a search method not included in the TREC pool.

### Text Collections

The results of the focused evaluation presented in Table 1 were performed by searching a no-title version of the whole Medline text collection consisting of about 16 million abstracts (the Medline baseline of 2006 was used). In order to compare with results obtained in the TREC Genomics Track, the high-recall evaluation was performed on the TREC-genomics track 2004 corpus (re-used in the 2005 evaluation). This corpus is a subset of Medline with about 4.5 million abstracts.

### Performance evaluations

The procedure *evaluate(H, D) *→ *P *is performed with the official TREC evaluation tool, trec_eval version 8.0 [19].

## Authors' contributions

FC designed the study, performed the experiments and wrote the manuscript.

## Supplementary Material

Additional file 1 Description of the second set of search methods used to prepare Figure 2. The file is in text format, tab delimited. Fields are described in the first line of the file. The file is in the format suitable to configure Twease for the specific search methods, but the information is similar to that shown in Table 3 of this manuscript (first set of search methods studied). Click here for file
